# Grain boundary passivation via balancing feedback of hole barrier modulation in HfO_2-*x*_ for nanoscale flexible electronics

**DOI:** 10.1186/s40580-022-00336-4

**Published:** 2022-09-30

**Authors:** Yeon Soo Kim, Harry Chung, Suhyoun Kwon, Jihyun Kim, William Jo

**Affiliations:** 1 New and Renewable Energy Research Center (NREC), Ewha Womans University, Seoul, 03760 Korea; 2 Department of Physics, Ewha Womans University, Seoul, 03760 Korea

**Keywords:** Flexible electronics, HfO_2-x_, Grain boundary, Passivation, KPFM

## Abstract

Flexible electronics has attracted considerable attention owing to its enormous potential for practical applications in various fields. However, the massive strain produced during bending degrades the device. Especially at grain boundaries, due to the accumulation of defects, this degradation is exacerbated in flexible electronic devices. The importance of electrically inactivated grain boundaries increases as devices scale down to the nanoscale. Here, we propose an HfO_2-*x*_ thin film that can be used as an excellent material for flexible electronics with versatile functionality, especially for grain boundary passivation. Various electrical phases of HfO_2-*x*_ thin films with conducting to insulating behavior, which originates from oxygen deficiency, have been fabricated on flexible substrates. Furthermore, owing to the most stable charge state of oxygen vacancies, oxygen-deficient HfO_2-*x*_ shows *p*-type conductivity. Current mapping by conductive atomic force microscopy reveals that current flow is hindered at grain boundaries due to the formation of potential barriers. This phenomenon is also observed in bent flexible thin films on convex and concave molds, leading to tensile and compressive strains, respectively. Although the defect concentration increases because of lattice deformation during bending, more holes are trapped at the grain boundaries, resulting in an increased hole barrier height. We believe that grain boundary passivation through hole barrier modulation during bending would pave the way for advances in hafnia-based nanoscale flexible electronics.

**Supplementary Information:**

The online version contains supplementary material available at 10.1186/s40580-022-00336-4.

## Introduction

Owing to its wide variety of applications, such as flexible circuits including displays, solar cells, sensor, and wearable devices, flexible electronics has attracted a great deal of interest as it can overcome the limited functionality of a rigid structure [1–4]. However, bending the device introduces severe failures such as cracks, dislocations, and grain boundaries, especially at grain boundaries where many defects, such as vacancies, accumulate to form local leakage current channels [5]. Furthermore, even in planar structures such as solar cells, defects accumulated at the grain boundaries degrade the device performance [6], but the degradation is accentuated in flexible electronic devices. As flexible electronics shrink to nanoscale dimensions, the significance of grain boundary passivation increases even more. Grain boundary passivation has various purposes: to reduce density of defect state/dangling bond, [7, 8] and to make electrically inactive [9]. Several passivation strategies were proposed to achieve efficient grain boundary passivation, such as post-annealing process/plasma treatment with various gases [8, 10], proper doping [11, 12], additional layers [13], highly oriented thin film growth technique [14]. However, these approaches inevitably require additional fabrication processes. In particular, high-temperature processes such as using a flexible metal substrate via ion-beam assisted deposition, strongly limit the use of flexible plastic substrates such as polyethylene terephthalate (PET) and polyethylene naphthalate (PEN). Here, we propose HfO_2_ as a promising candidate for flexible electronics based on spontaneous grain boundary passivation, in which potential barriers are naturally formed at grain boundaries, leading to grain boundary electrically inactive.

HfO_2_ is widely used as a gate dielectric in metal oxide semiconductors, which are an alternative to SiO_2_, owing to its high dielectric constant (*ε*_r_ ~ 25), wide bandgap (~ 5.7 eV), and high thermal stability, which has already been successfully introduced into fabs instead of SiO_2_ because it allows for minimized cross-contamination [15–17]. Furthermore, ferroelectricity induced in the fluorite phase of HfO_2_ by using appropriate dopants is of considerable interest since it can be used to form a ferroelectric tunnel junction or give rise to negative differential capacitance and thereby increase the subthreshold swing over 60 mV/decade in a field-effect transistor [18]. It is owing to these robust functionalities and complementary metal–oxide–semiconductor-compatible properties, which have led to it being in high demand in the conventional rigid semiconductor industry as well as flexible electronics industry. HfO_2_-based flexible electronics have been intensively investigated for high-*k* gate dielectrics as well as resistive switching behaviors [19–22]. In the flexible electronics, grain boundary passivation is essential to prevent unintended grain boundary mediated-dielectric breakdown during bending. This is especially crucial in HfO_2_-based flexible neuromorphic devices, as oxygen deficiency variation of conductive channels is accountable for a gradual change of conductance [23]. For fabrication of the hafnia-based flexible electronics, deposition techniques for large-area growth such as atomic layer deposition and sputtering are commonly used [19]. These large-area growth techniques inevitably form amorphous-like polycrystalline HfO_2_ thin films containing various defects, such as grain boundaries and oxygen vacancies. However, not all oxygen vacancies are defects that need to be eliminated, and they have a variety of functionalities. Oxygen-deficient clusters often form conductive channels, and the formation/rupture of the channels through the application of an external bias is the basis of resistive switching in numerous metal oxides [21, 24]. In particular, on the basis of density functional theory calculations, a previous study predicted compensated tetragonal semimetallic Hf_2_O_3_ suboxide as the ground state of highly oxygen-deficient hafnium oxide [25]. Therefore, to examine the effect of oxygen deficiency in the flexible hafnium oxide thin films, we will show various phases corresponding to different electrical behavior ranging from conducting to insulating behavior by carefully adjusting growth conditions.

## Results and discussion

**Fig. 1:**
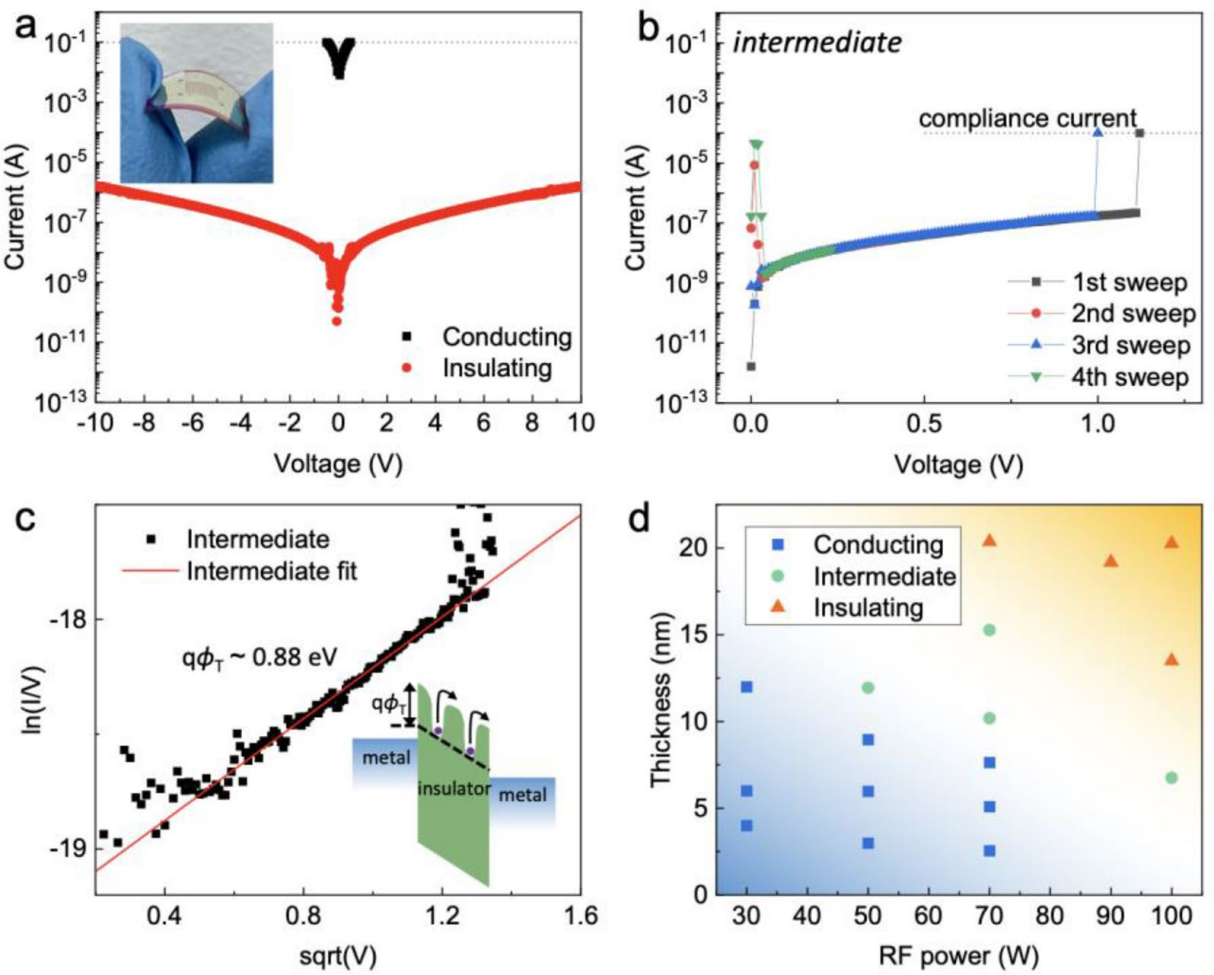
Electrical behavior of HfO_2-*x*_ thin films in various electrical phases. **a** *I-V* characteristics of the conducting (black) and insulating (red) states. The inset shows a photograph of a flexible device. **b** Typical unipolar resistive switching behavior in the intermediate states. **c** Poole–Frenkel fitting result for the HRS for the intermediate states. The inset shows a schematic of carrier hopping in the Poole–Frenkel conduction. **d** Phase diagram showing various phases as functions of thickness and rf power

In this study, HfO_2-*x*_ thin films fabricated on the flexible Ag/PEN substrate under various conditions using a sputtering method, which provides various deposition conditions. As a flexible substrate, lower glass transition temperature of PET (T_g_ ~ 78 °C) than of PEN (T_g_ ~  °C) imposes a limitation on its permissible operating temperature [26]. The low glass transition temperature may influence the surface morphology, which is detrimental to the investigation of effect at grain boundaries [27]. The various conducting states of HfO_2-*x*_ thin films could be categorized into three types based on their electrical behavior: conducting, insulating, and intermediate electrical behavior. Figure 1a–c shows the *I-V* characteristics of the HfO_2-*x*_ thin films. The typical conducting and insulating phases are shown in Fig. 1a, and their bending feature is shown in the inset of Fig. 1a. Furthermore, the intermediate phase between the metallic and insulating phases showed highly metastable electrical behavior. Unlike the insulating phase, the intermediate phase generally showed unstable electrical behaviors and occasionally exhibited typical unipolar resistive switching, as shown in Fig. 1b. During resistance switching from the high resistance state (HRS) to the low resistance state (LRS) by the formation of conducting filament, referred to as the set process, the compliance current was set to 0.1 mA to prevent the permanent breakdown of the thin films. Upon the repeated application of a bias voltage, the resistance changed from the LRS to the HRS by the rupture of the conducting filament, which is referred to as the reset process. Although the unipolar resistive switching shows poor endurance behavior (< 50 cycles), the repetitive resistive switching was performed at a relatively low voltage with a low reset current. In general, the reset process in unipolar resistive switching requires a high reset current to accompany Joule heating, but unipolar resistive switching in the intermediate phase could be performed with a relatively low reset current.

To elucidate the conduction mechanism in the HRS, we theoretically determined the transport characteristics in the HRS. The conduction behavior was dominated by the bulk-limited Poole–Frenkel emission: [28].

1 $$J=q\mu {N}_{C}E exp\left(\frac{-q\left({\phi }_{T}-\sqrt{\frac{qE}{\pi }{\varepsilon }_{i}{\varepsilon }_{0}}\right)}{{k}_{B}T}\right)$$

where *J* is the current density, *q* is the electric elementary charge, *N*_*C*_ is the density of states in the conduction band, *μ* is the carrier mobility, $q{\phi }_{T}$ is the trap energy, *ε*_0_ is the permittivity of free space, *ε*_i_ is the relative dielectric constant of HfO_2_, *k*_B_ is the Boltzmann constant, *T* is the temperature, and *E* is the electric field. In the presence of an electric field, the potential energy gradient may increase the probability of thermally assisted hopping of electrons between localized traps, as depicted in the inset of Fig. 1c. According to Eq. (1), a plot of $ln\left(\frac{I}{V}\right)$ *vs*. $\sqrt{V}$ is linear, as shown in Fig. 1c. Furthermore, the trap energy can be extracted as a fitting parameter from the linear relationship. The value obtained in this study was about 0.88 eV, which was similar to a previous result [29].

The lower the sputtering power and the smaller the film thickness, the greater the possibility of the conducting phase appearing. On the other hand, the higher the sputtering power and the larger the thickness, the greater the possibility of the insulating phase being observed. Figure 1d shows the various phases as a function of the sputtering power and thickness. The velocity of sputtered HfO_2-*x*_ increases with the rf power, and a higher velocity leads to a longer mean free path with a lower chance of oxygen loss through collisions of the sputtered atoms with Ar gas molecules in the sputtering chamber [30], as shown in Fig. S1. The scalar *s* and the mean free path in units of centimeters are given by [30].

2 $$s=107.7242\sqrt{\frac{E [eV]}{T [K]}}\sqrt{\frac{{M}_{Ar}}{{M}_{Hf}}}$$

3 $${\lambda }_{proj}[cm]= \frac{s}{\left[(s+\frac{1}{2s})erf(s)+\frac{1}{\sqrt{\pi }}exp(-{s}^{2})\right]}\cdot \frac{3.297cm\bullet T[K]}{{({R}_{Hf}[pm]+{R}_{Ar}[pm])}^{2}{P}_{Ar}[mbar]}$$

where M, R, and P denote mass, ionic radius, and partial pressure, respectively. Note that the resistance of the samples did not scale with the thickness and indicated the nonuniformity of the samples, which strongly suggested the presence of local conductive channels. The channel could have comprised oxygen vacancies associated with oxygen-deficient Hf suboxide [24], or it could have been an Ag conductive filamentary channel [31]. However, resistive switching caused by Ag ion migration should exhibit bias polarity dependence, that is, a negative bias for attracting Ag^+^ ions and a positive bias for repelling of Ag^+^ ions. In view of the repeated resistance changes with a bias of a single polarity, the conductive channel could not have comprised an Ag filamentary channel.

**Fig. 2:**
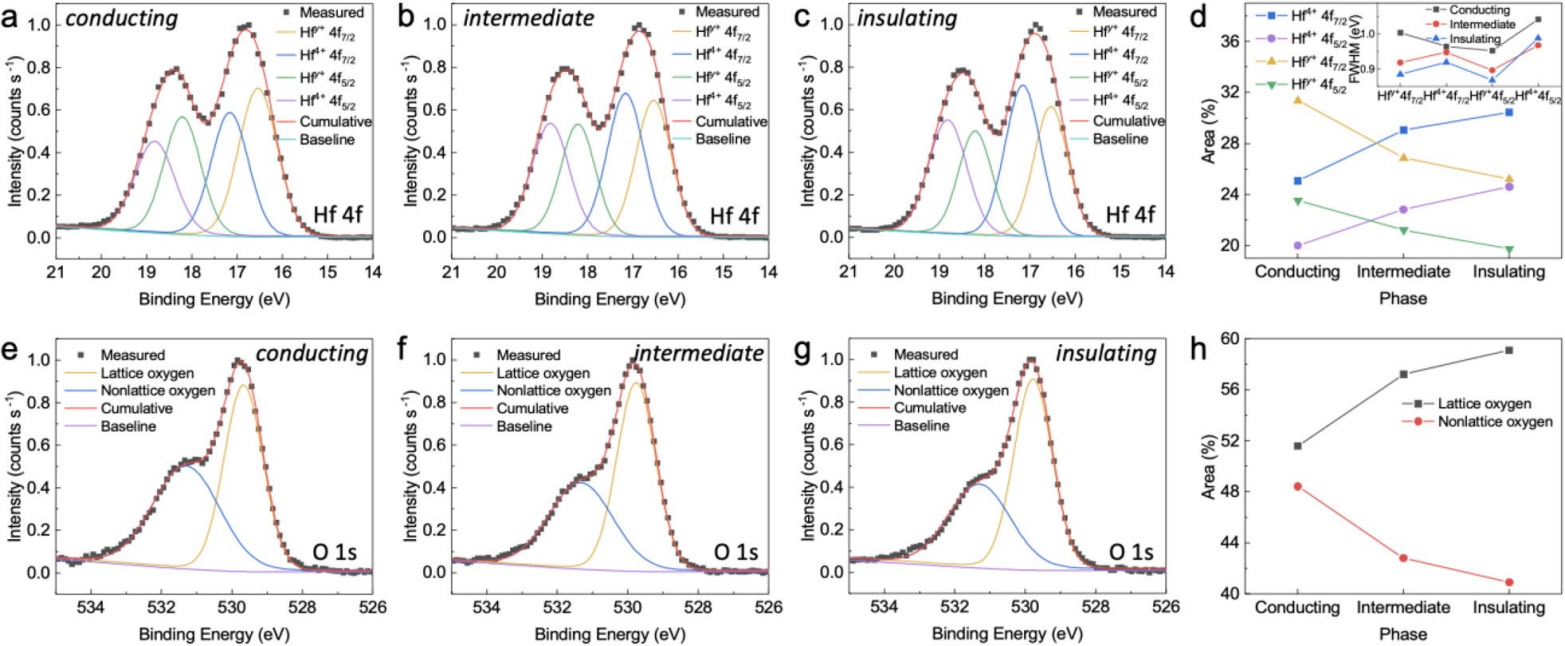
XPS spectra of HfO_2-*x*_ thin films in various electrical phases. The fitting results for the Hf 4f spectra of the **a** conducting, **b** intermediate, and **c** insulating states are presented. **d** The areas of stoichiometric Hf^4+^ and oxygen-deficient Hf^*y*+^ spectra in different states. The inset shows the FWHM of each corresponding deconvoluted spectra. O 1 s spectra of the **e** conducting, **f** intermediate, and **g** insulating states are also shown. **h** The areas of lattice oxygen and nonlattice oxygen are in different states

X-ray photoelectron spectroscopy (XPS) spectra of Hf 4f for the conducting, intermediate, and insulating phases are shown in Fig. 2a–c. The Hf 4f spectrum could be well fitted with Hf^4+^ and Hf^*y*+^ (*y* < 4) with double peaks. It is corresponded to stoichiometric HfO_2_ and Hf suboxide, respectively, which is the widely accepted for oxygen-deficient HfO_2-x_ [32–35]. Furthermore, the Hf^4+^ 4f_7/2_ and Hf^4+^ 4f_5/2_ doublet peaks were located at 17.16 and 18.82 eV, respectively, while the Hf suboxide doublet, namely Hf^*y*+^ 4f_7/2_ and Hf^*y*+^ 4f_5/2_, was located at lower binding energies of 16.54 and 18.32 eV, respectively. The location of Hf suboxide peaks was observed at a much lower binding energy [36–38], Hf suboxide peaks are significantly reduced in the relatively stoichiometric HfO_2_ thin film, which grown in a relatively oxygen-rich environment (Ar: O_2_ = 10: 2) in Additional file 1 Fig. S2. This result strongly demonstrates that the peaks at 16.54 and 18.32 eV are highly related to Hf suboxide. The change in stoichiometry could be assessed from the change in the area of the corresponding peaks. Interestingly, the HfO_2-*x*_ thin film exhibited more insulating behavior as the ratio of the Hf^4+^ peak area to the Hf^*y*+^ peak area increased, as shown in Fig. 2d, implying that this ratio of Hf suboxide strongly influenced the compound’s electrical behavior. Furthermore, as demonstrated in the inset of Fig. 2d, a higher full width at half-maximum (FWHM) of more conductive HfO_2-x_ thin films implies the existence of a higher degree of disorder due to oxygen vacancies in the thin films [32, 39].

Figure 2e–g shows O 1 s core-level spectra of the conducting, intermediate, and insulating phases. The prominent peaks in the spectra associated with lattice oxygen could be attributed to HfO_2_ and they can be observed at 529.8 eV, and additional broad peaks related to nonlattice oxygen are apparent at 531.3 eV, and they are associated with OH^−^ or an oxygen vacancy [32]. The nonlattice oxygen peak with lower intensity for more insulating phases is associated with Hf suboxide, which indicates the nonlattice oxygen is corresponding electrical conductivity [32]. Also, the non-lattice oxygen peak area remarkably reduced in the relatively stoichiometric HfO_2_ thin film in Additional file 1 Fig. S2. Although considering the area of nonlattice oxygen peak still remains about 31%, mainly due to the presence of hydroxyl group, the significant decrease in the nonlattice oxygen peak indicates that oxygen vacancies are also strongly involved in the nonlattice oxygen peak. Figure 2h shows a relatively large portion of the nonlattice oxygen peak even in the insulating phase since the deposition process was performed in an oxygen-deficient environment. Additionally, although there are slight differences (within several percent) in the areas of nonlattice oxygen peaks, it shows significant differences in their electrical properties that range between conducting and insulating phases with similar to the percolative threshold [40], as shown in Fig. S3. This result strongly indicates that local electrical properties play a critical role in determining the overall electrical properties.

**Fig. 3:**
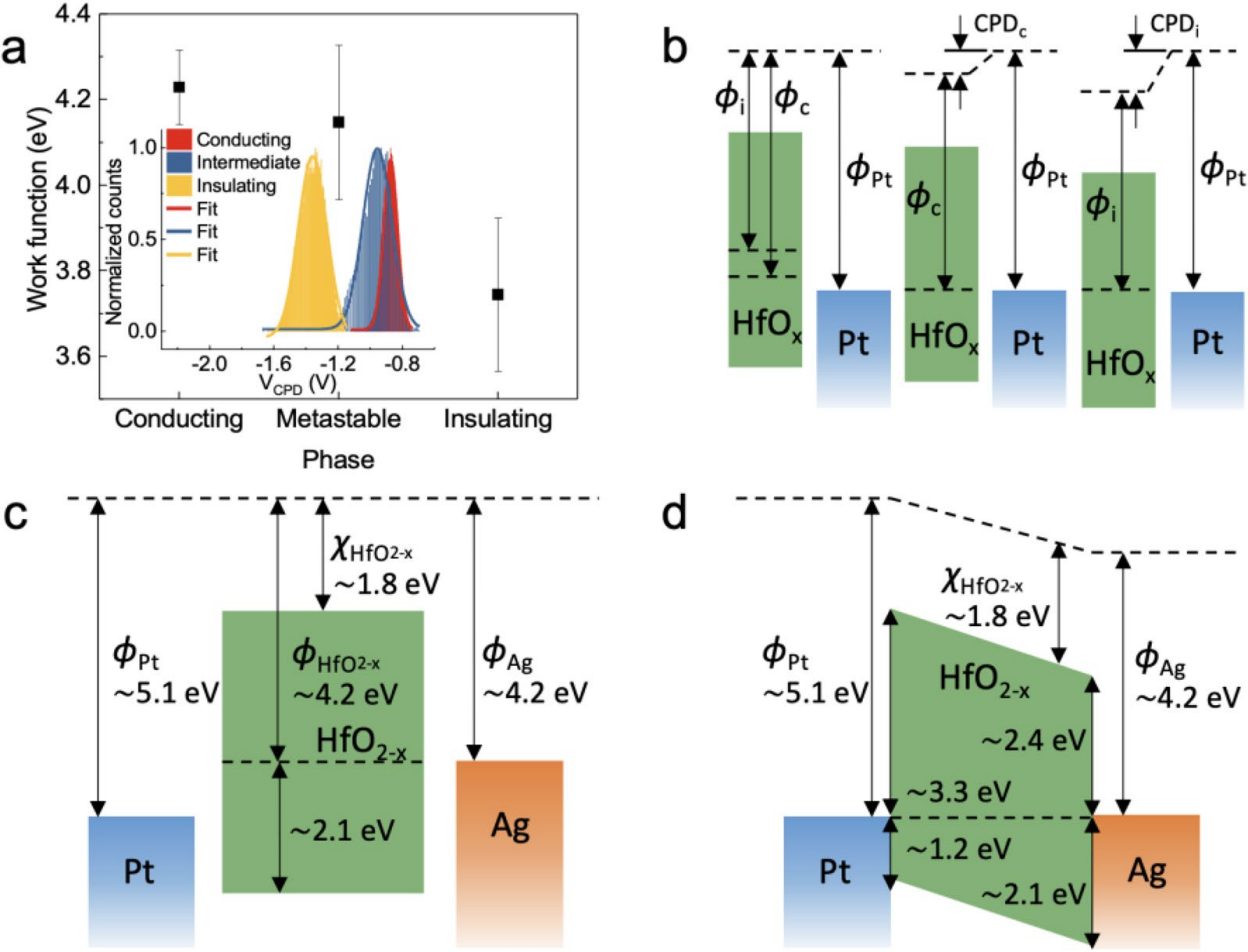
**a** Work function modification for various electrical phases of HfO_2-*x*_. The inset shows histograms of CPD maps. **b** Schematics of different work functions with different CPDs in the conducting and insulating states (denoted by c and i, respectively). Band diagrams of Pt/HfO_2-x_/Ag structure of **c** before and **d** after contact

For the further investigation of the surface potential of the conducting, intermediate, and insulating phases, topographies and surface potential map measurements were obtained using Kelvin probe force microscopy (KPFM), and they are shown in Additional file 1 (Fig. S4a–c), respectively. The inset of Fig. 3a shows the histogram of each contact potential difference (CPD) distribution obtained from the corresponding maps in Fig. S4a–c. From the CPD values, the work function of samples (${\Phi }_{sample}$) can be calculated using the equation

4 $${\Phi }_{sample}={\Phi }_{tip}-e{V}_{CPD}$$

where ${\Phi }_{tip}$ is the work function of the tip, and *e* is the elementary charge. The calculated work functions are shown in Fig. 3a; the insulating phase had the smallest work function, while the conducting phase had the largest work function, which provides strong evidence that HfO_2-*x*_ had *p*-type conductivity. The *p*-type conductivity has been reported elsewhere [41–43]and is believed to result from oxygen vacancies primarily. Note that the XPS results show that the conducting phase contains the most abundant oxygen vacancies. The charge state of oxygen vacancies is generally formed as V_O_^2+^ acts as a donor. However, on the basis of density functional theory calculations, Broqvist et al. [29] reported that fourfold coordinated V_O_^−1^ is the most stable defect state in HfO_2_, and that the state as the physical origin of the observed trapping center was below about 0.9 eV from the conduction band edge. This value is close to the trap energy of about 0.88 eV calculated from the Poole–Frenkel fitting results in Fig. 1c. As evident in Fig. 3b, the large CPD in the insulating phase leads to a small work function, while the small CPD in the conducting phase results in a large work function because of the fourfold coordinated V_O_^−1^ state acting as an acceptor. It is essential for understanding their electrical characteristics by estimating exact Fermi energy with respect to valance- and conduction bands. However, owing to the bandgap of HfO_2-x_ severely varies from 4.5 eV to 5.6 eV by addressing the amount of oxygen vacancies, it is difficult to define the exact energy levels of the conduction band, valence band, and Fermi level of oxygen-deficient HfO_2-x_. Therefore, assuming that dielectric permittivity of HfO_2_, ${\varepsilon }_{r}$, built-in potential, ${V}_{bi}$, the band gap, electron affinity of conducting HfO_2-x_ is 25 [15], 0.9 eV, 4.5 eV [41], 1.8 eV [44], respectively. Also, considering that the resistance of the conducting HfO_2-x_ thin film is several ohms to several tens of ohms, it could be estimated that the doping concentration, ${N}_{A}$, is as high as that of the highly-doped semiconductor, which is about 1✕10^19^ cm^−3^. Its depletion layer width, ${W}_{D}$, is about 16 nm by using the equation, ${W}_{D}=\sqrt{2{\varepsilon }_{0}{\varepsilon }_{r}{V}_{bi}/{qN}_{A}}$, the depletion layer is larger than its thickness. Therefore, as shown in Fig. 3c, d, its band diagram represents the *p*-type nature of oxygen-deficient HfO_2-x_. In addition, the effect of the surface oxide layer should be taken into account. The surface passivation effect by a few nm-oxide layer often plays a dominant role in their electrical effect on the entire thin film [45]. However, the large difference of surface potential corresponding electrical behaviors between the conducting, intermediate, and insulating phases are difficult to explain by the surface oxide layers. Also, due to the probing depth of the Al-Kα source in XPS being within ~ 5 nm beneath the surface [46], XPS spectra should be the same with the presence of the surface oxide layer.

**Fig. 4:**
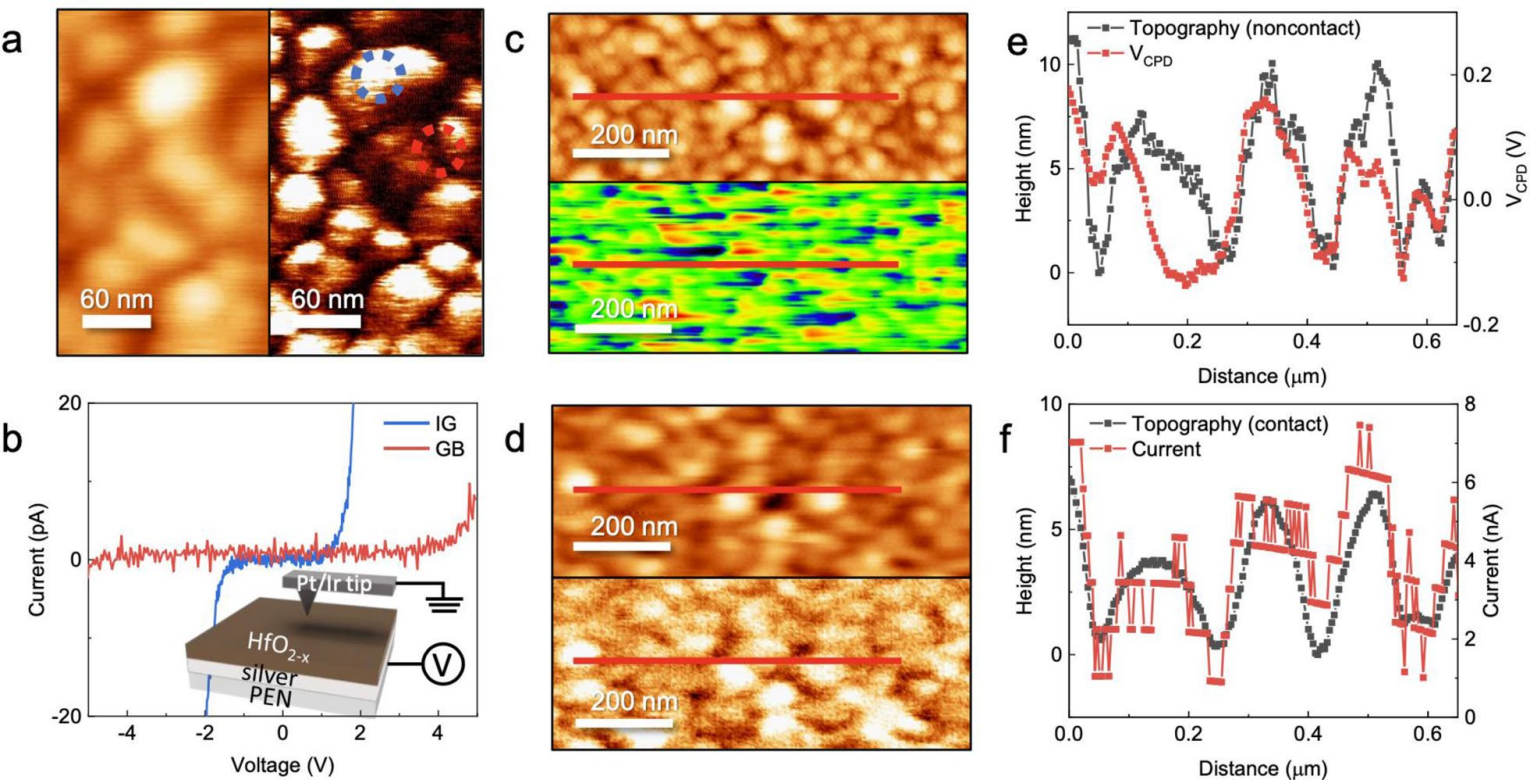
CPD and current mapping corresponding topography for a HfO_2-*x*_ thin film. **a** Simultaneously obtained topography (left) and current mapping images (right) for the conducting states. The blue and red dotted circles represent intergrain and grain boundaries, respectively. **b** Local *I-V* data for the intergrain (blue) and grain boundaries (red). The inset shows a schematic of the cAFM setup used for characterizing HfO_2-*x*_ thin films. **c** Topography (top) and CPD mapping image (bottom) and **d** topography (top) and current mapping image (bottom) at the same position for the conducting states. Line profiles of **e** topography and CPD mapping images and **f** topography and current mapping images along the red lines in (**c**) and (**d**)

For the investigation of the relationship between morphology and local conductivity, atomic force microscope (AFM) and conductive atomic force microscopy (cAFM) measurements were conducted. Figure 4a shows topography images and corresponding cAFM images of the conducting phase. Clearly, the local current flows preferentially at the intergrain rather than at the grain boundary. Figure 4b shows local *I-V* plots at the intergrain and grain boundaries, and the two boundaries are marked by blue and red dotted circles in Fig. 4a, respectively. An external bias was applied to sample bias, as shown in the inset of Fig. 4b, while the AFM tip was grounded. While relatively insulating behavior was observed in the grain boundary region, relatively metallic behavior was evident in the intergrain region, suggesting the formation of depletion regions at grain boundaries. Furthermore, the local *I-V* behavior appeared symmetric at the intergrain and grain boundaries, consistent with the *I-V* characteristics in Fig. 1. This behavior shows that carrier transport was dominated by bulk-limited conduction rather than by interface-limited conduction despite the significant difference in the work function between the Pt/Ir AFM tip and Ag bottom electrode [47, 48]. Fig. S5 shows topography images and the corresponding cAFM images of the conducting (Fig. S5a, d), intermediate (Additional file 1: Fig. S5b–e), and insulating phases (Additional file 1: Fig. S5c–f). The topography images obtained for external sample bias voltages up to 1.5, 6, and 10 V for the conducting, intermediate, and insulating phases, respectively, do not show any significant surface morphology change, implying that oxidation/hydrogenation of the HfO_2-*x*_ thin films does not affect their resistivity significantly [49]. Additional file 1: Fig. S5d–f shows the current mapping for various applied sample bias voltages for the conducting, intermediate, and insulating phases, respectively. While no conductive region is visible in the intermediate and the insulating phases for sample bias voltages up to 6 and 10 V, respectively, conductive channels begin to appear over 1 V in the conducting phase in Fig. S5(d). Note that the local current flows preferentially at intergrain rather than grain boundaries, even for the negative bias. Although surface morphology often plays a vital role for electrical transport, the more insulating sample has a higher root-mean-square of roughness, as shown in Additional file 1 Fig. S5g. Also, electrical current preferentially flows through relatively thick intergrains rather than grain boundaries. Therefore, local morphology might limitedly affect their electrical properties. This observation also supports the existence of dominant bulk-limited conduction at the intergrain and grain boundaries.

In a polycrystalline structure, the grain boundary shows significantly different electrical properties compared with the intergrain structure, and it independently contributes to electrical conduction. For the examination of their electric potential, corresponding morphology, and local conductivity, cAFM measurements, which can obtain current mapping signals, and CPD maps were obtained for the same area for the conducting phase. Figure 4c shows topography (top) and CPD mapping images (bottom) obtained simultaneously for the conducting phase by using KPFM measurement, and Fig. 4d shows topography (top) and current mapping at 1 V (bottom). Although Fig. 4d shows a blurred image because of the damaged AFM tip, the position is apparently the same as that in Fig. 4c. From the simultaneously acquired topography and CPD mapping images, image profiles along the red lines extending across several grain boundaries in Fig. 4c were extracted, shown in Fig. 4e. Interestingly, lower CPD was observed at the grain boundaries. It indicated that the charge distribution differed between grain boundaries and intergrain and that downward band bending occurred at grain boundaries [11]. Since the conducting HfO_2-*x*_ thin film had *p*-type conductivity, the downward band bending prevents current flows. Figure 4f shows the red line profile of the topography and current mapping image at the same position in Fig. 4c, d.

Typically, leakage current paths form at grain boundaries where V_O_^2+^ accumulate and act as a donor in the oxide. However, in *p*-type oxygen-deficient HfO_2-*x*_, the most stable oxygen vacancy is V_O_^−1^, which acts as an acceptor [29, 50]. The downward band bending occurs because of hole trapping [11], and it forms a depletion region at the grain boundary and leads to grain boundaries inactive electrically. This spontaneous passivation effect is entirely beneficial for flexible electronic devices. In general, repeated tensile and compressive deformations by bending flexible electronic devices give rise to many defects, such as vacancies mostly at grain boundaries. The deformation and defects result in the formation of leakage paths and vacancy build-up, which cause a dielectric breakdown. Therefore, it is imperative to investigate the effect of grain boundary passivation in bent flexible thin films.

**Fig. 5:**
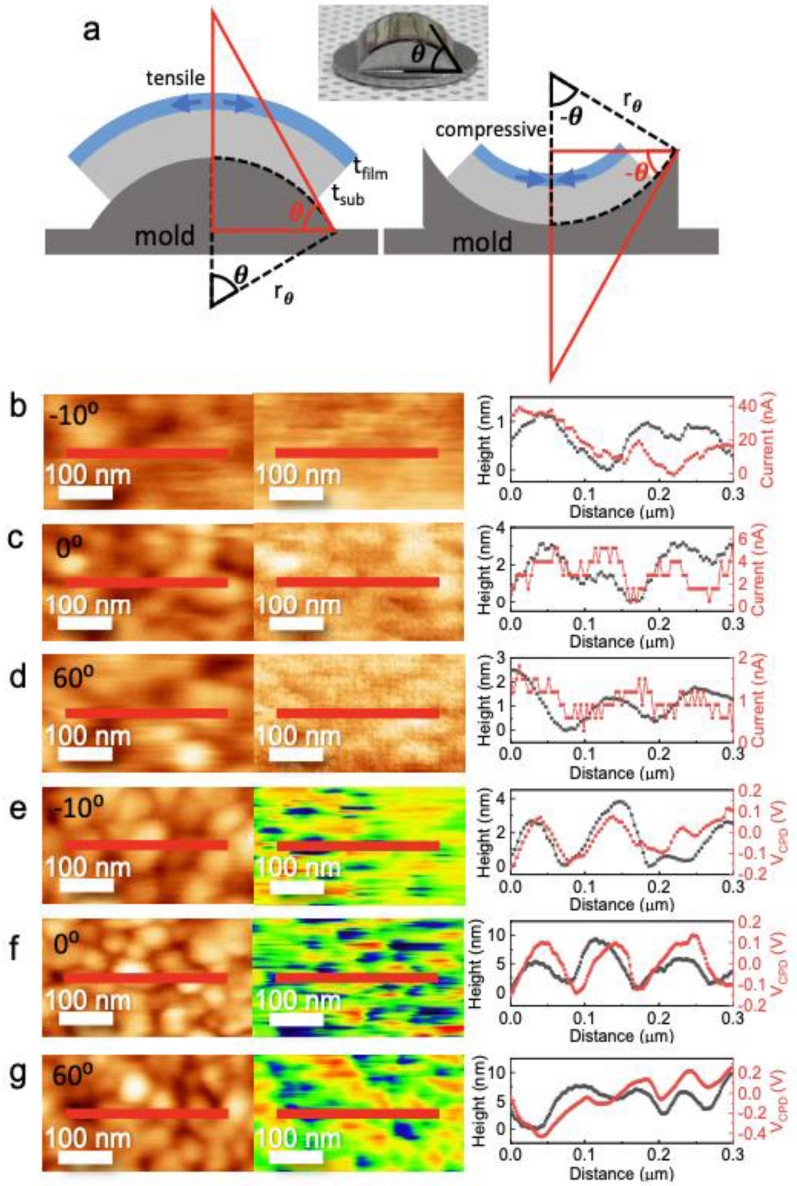
Grain-boundary-related CPD and current mapping for the conducting HfO_2-*x*_ thin film during bending. **a** Schematics of flexible thin films on convex (left) and concave (right) molds and the corresponding tensile and compressive strain, respectively. A photograph of the bent HfO_2-*x*_ thin film is shown in the middle. Topographies (left) and current mapping images (middle) obtained at **b** -10°, **c** 0°, and **d** 60°, and topographies (left) and CPD mapping images (middle) obtained at **e** -10°, **f** 0°, and **g** 60°. Line profiles (right) are shown for the red lines

For the determination of the bending properties of flexible HfO_2-*x*_ thin films, convex and concave molds of various angles were fabricated; the molds are schematically shown in Fig. 5a and Additional file 1 (Fig. S6). When a flexible HfO_2-*x*_ thin film was placed in a convex and concave mold, as shown in the inset of Fig. 5a, tensile and compressive strains were induced in the thin film, as shown in Fig. S6. The applied strain is

5 $${r}_{\theta }=d\theta$$

6 $$\varepsilon \left(\%\right)=\frac{\left({r}_{\theta }+{t}_{sub}+{t}_{film}\right)\theta -{r}_{\theta }\theta }{{r}_{\theta }\theta }\times 100$$

where $\theta$ is the angle, ${r}_{\theta }$ is the radius, *d* is the half-chord, and *ε* is the strain applied to the thin film. Positive and negative signs represent tensile and compressive strains, respectively. To investigate the microscopic properties at the grain boundaries in the bent thin films, we obtained the CPD and current maps of the flexible HfO_2-*x*_ thin films on convex and concave molds. Figure 5b–d shows topographies and the corresponding cAFM images of the mold for angles of −10°, 0°, and 60°, respectively. Because the AFM head unit was contacted with the mold, we could not measure using concave molds with less than −10°. The negative angle indicates the concave mold, as shown in Additional file 1: Fig. S6c. The calculated curvature radius and corresponding strain as a function of angles is represented in Additional file 1: Fig. S6d. The red line profiles clearly showed that the current preferentially flowed at intergrain, which is consistent with observations for planar structures. Figure 5e–g shows topographies and the corresponding CPD mapping images of the mold for the angles −10°, 0°, and 60°, respectively. Although CPD and current mapping were measured at different regions, lower CPDs were observed at grain boundaries as well. In particular, note that the grain boundary passivation effect is observed in the bent samples, implying that HfO_2-x_ is an ideal material for flexible electronic devices.

**Fig. 6:**
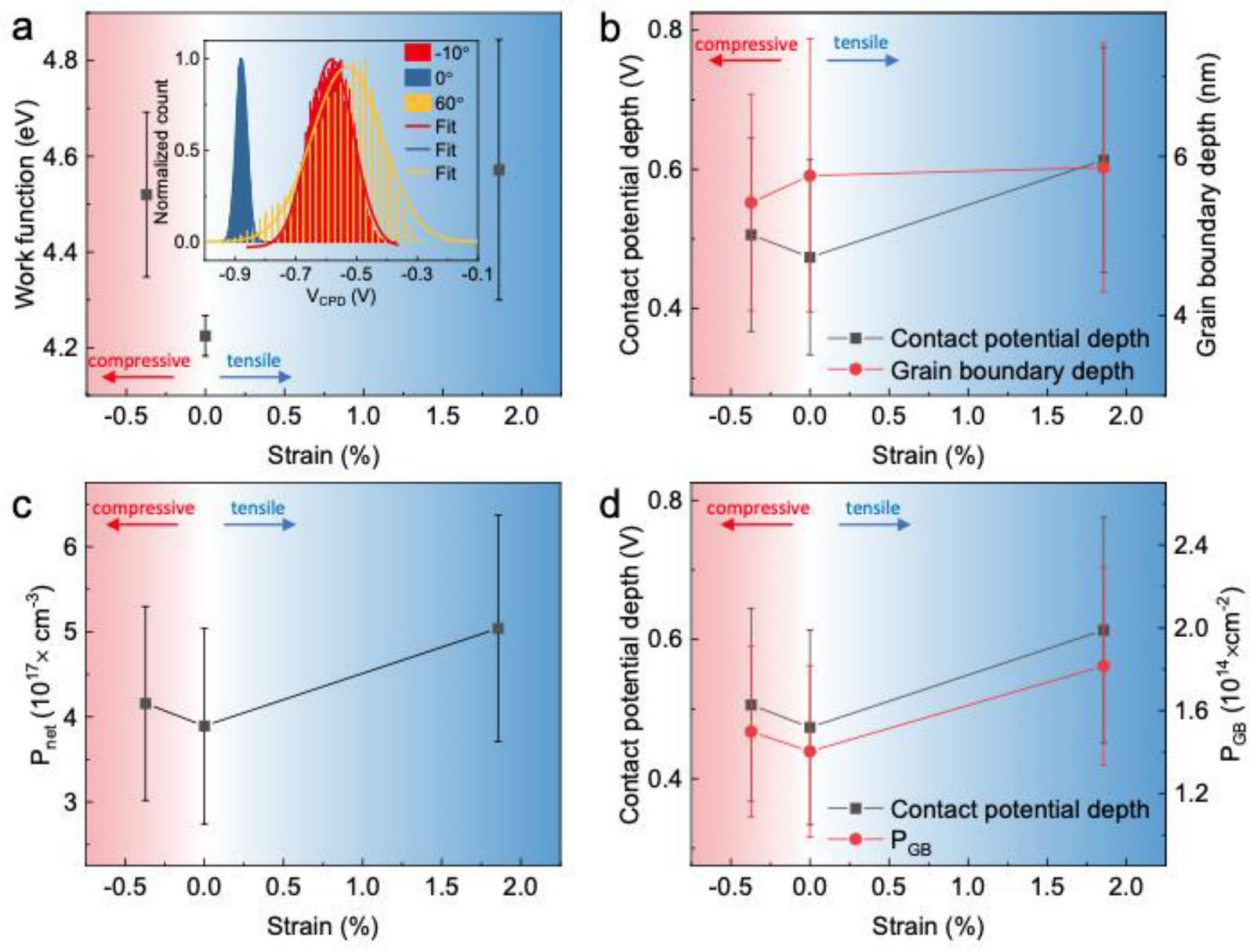
Grain boundary passivation effect through balancing feedback of hole barrier modulation. **a** Work function modification under various strains in HfO_2-*x*_. The inset shows histograms of CPD maps. Variation of **b** the CPD depth (black) and grain boundary depth (red), **c** the net doping density, *P*_*net*_, and **d** the CPD depth (black) and defect density (red) at the grain boundary as a function of the strain

The inset in Fig. 6a shows the change in the work function with bending. The lowest value of the work function was observed for the planar structure, and the value increased with both applied tensile and compressive strains, as shown in Fig. 6a. This result implies that both the tensile and compressive strains give rise to a *p*-type doping concentration, which is consistent with the previously reported result that lattice deformation by biaxial strain results in lower formation energy of oxygen vacancy in HfO_2_.[51] In order to examine the relationship between morphology and CPD, we measured the amplitude between the trough and crest in the topographies and CPD maps, as the red and green crosses marked in Additional file 1: Fig. S7. The grain boundary depth in morphology does not significantly affect the surface potential, as shown in Fig. 6b. However, the contact potential depth is increased under both tensile and compressive strains present, and the hole barrier height increases because of more trapped holes at the grain boundaries. Furthermore, the increase in the trapped hole concentration reinforced the grain boundary passivation even in the bent HfO_2-*x*_ thin film. The number of trapped charges at the grain boundary can be estimated based on the Poisson equation and the Schottky approximation: [52, 53].

7 $$\varepsilon {\varepsilon }_{0}\frac{{\partial }^{2}{V}_{GB}(l)}{\partial {l}^{2}}=-\rho \left(l\right)=-e{P}_{net}$$

8 $$\Delta {V}_{GB}(l=0)=\frac{e{P}_{net}}{2\varepsilon {\varepsilon }_{0}}{w}^{2}$$

9 $${P}_{GB}=\sqrt{\frac{8\varepsilon {\varepsilon }_{0}{P}_{net}\Delta {V}_{GB}}{e}}$$

where $\varepsilon$ is the dielectric constant of HfO_2_, ${\varepsilon }_{0}$ is the dielectric constant of vacuum, *V*_*GB*_ ($l$) is the potential distribution across the grain boundary, $l$ is position between 0 (at grain boundary) and *w* (width of space charge region), *P*_*net*_ is the net doping density of the HfO_2-x_ thin films, *P*_*GB*_ is the defect density at the grain boundary. The increased net doping density during bending is shown in Fig. 6c. As can be clearly seen in Fig. 6d, the increase in defect density with increasing contact potential depth is due to the increase in ionized trapped hole density at the grain boundary during bending [52], the increase of trap density induced by bending is observed elsewhere [54]. In addition, we have to consider that repeated bending may affect to band structure. To identify variations in work function, additional CPD measurements were conducted during bending. As indicated by additional work function measurements as shown in Additional file 1: Fig. S8a, we were unable to observe a significant change in the Fermi level. Therefore, we can anticipate that repeated bending would not significantly affect the band structure. Besides, to investigate reproducibility and stability of the gain boundary passivation effect the variation in contact potential depth and hole trap density at grain boundaries as a function of bending cycles was investigated. The absence of significant variation in the trap densities indicates that the grain boundary passivation is unaffected by up to 500 cycles of bending in planar structure as well as in a bent structure with the mold of 60° angle, as illustrated in Additional file 1: Fig. S8b, c. Macroscopic resistance change as a function of strain also does not exhibit significant change as illustrated in Additional file 1: Fig. S9a. Further, we measured the resistance of the conducting and insulating phases with an increase in the bending cycle with the angle of about ± 60°, and both resistances did not change up to 500 cycles, as shown in Fig. S9(b). In addition, we could not observe any surface deformation after several hundred bending cycles. In general, macroscopic defects, such as microcracks and delamination, are primarily caused by poor adhesion [55], and a strong adhesion between the substrate and the thin film formed by energetic particles during sputtering would prevent the occurrence of macroscopic defects. Although unipolar resistive switching behavior in the intermediate phase does not show good reliability, bistable resistive switching behaviors showed up to 500 times of bending cycles (Additional file 1: Fig. S9c and d).

**Fig. 7:**
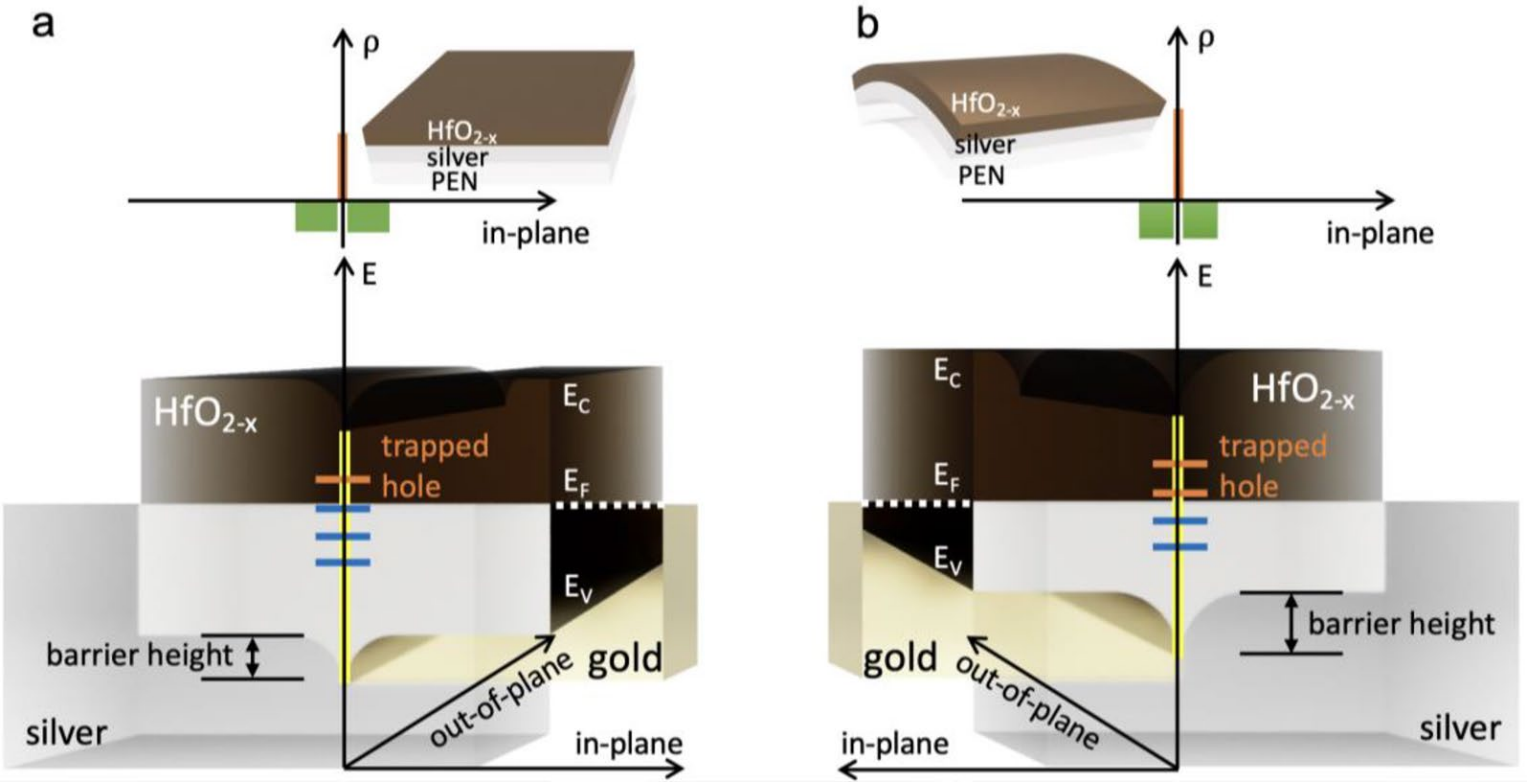
Band structure differences between **a** a planar and **b** a bent HfO_2-*x*_ thin film. The space charge distribution (top) represents a thermal equilibrium state between the trapped hole density at the grain boundary (orange) and the negatively charged acceptor density (green) in a cross section across grain boundaries in the in-plane direction. The insets in **a** and **b** show a planar and a bent HfO_2-*x*_ thin film, respectively. In the three-dimensionally expressed band diagrams (bottom), grain boundaries are marked by yellow lines, and the blue and orange lines on the grain boundaries indicate electron-filled and trapped hole states, respectively. In particular, the contact planes between HfO_2-x_ and silver of the band diagram are depicted with the color of silver

Figure 7a, b show the band structure of the planar and bent conducting HfO_2-*x*_ thin films obtained from our experiments, respectively. The conducting phase exhibited a *p*-type band structure because of a large number of oxygen vacancies, as shown in Fig. 7a. Additionally, oxygen vacancies were clustered due to the low formation energy at the grain boundaries [56], where a large number of holes were trapped in the abundant trap sites. Figure 7b depicts the band structure of the bent HfO_2-*x*_ thin films. The doping concentration increased following lattice deformation, and an increase in the trapped hole density decreased the negatively ionized oxygen vacancy concentration. A comparison of the band diagrams for the insulating, conducting, and bent conducting phases is shown in Additional file 1: (Fig. S10). In the insulating phase, a relatively small number of holes were trapped at the trap sites at the grain boundaries owing to the small number of acceptors in thermal equilibrium in Additional file 1: Fig. S10a. A lower doping concentration resulted in a higher work function (Fig. 3a), which led to an increase in the hole barrier height. Note that an increase in the doping concentration resulting from an increase in the defect concentration because of bending might increase the hole barrier height and obstruct the flow of electric current at the grain boundaries. The balancing feedback associated with spontaneous grain boundary passivation due to hole trapping at the grain boundaries plays a critical role in flexible electronics.

Notably, the grain boundary passivation contradicts previous results, according to which a local current flows preferentially at the grain boundaries of an HfO_2_ thin film [57–59]. Common device structures in previous results involved electrodes with a high work function, such as *p*-Si (ɸ ≈ 4.91 eV) [60] or TiN (ɸ ≈ 4.7 to 4.9 eV) [61], and Ohmic contact was maintained at the interface between grain boundaries and the bottom electrode. However, in the experiment performed in the current study, a silver electrode (ɸ ≈ 4.26 eV)[47] was used, and a potential barrier was formed at the interface. Therefore, for flexible electronics involving spontaneous grain boundary passivation in HfO_2-x_, a material with a low work function is necessary.

## Conclusions

In this paper, oxygen-deficient HfO_2-*x*_ thin films were deposited on a flexible silver/PEN substrate. The thin films showed various electrical phases: conducting, intermediate, and insulating phases. XPS analysis showed that these phases had different Hf suboxide ratios. Furthermore, CPD and current mapping measurements revealed the *p*-type semiconducting behavior of oxygen-deficient HfO_2-*x*_, which resulted from the stable V_O_^−1^ oxygen vacancy acting as an acceptor. Interestingly, spontaneous grain boundary passivation was observed, obstructing current flow at the grain boundaries. It is particularly beneficial for nanoscale flexible electronics, where grain boundaries become increasingly essential. In addition, in CPD and current mapping measurements, it has been observed to increase the hole barrier height when the concerned device is bent. We believe that these results have the potential to contribute to the development of flexible electronic devices for hafnia-based applications.

## Experimental section

### Device fabrication

We deposited 50 nm thick Ag as the bottom electrode on a flexible PEN substrate through thermal evaporation at room temperature prior to the growth of HfO_2-*x*_ thin films. Thickness of PEN is 150 µm (Teijin Dupont Films). Before the deposition of the Ag bottom electrode, the PEN substrate was ultrasonically cleaned with acetone, ethanol, and deionized water for 10 min, in this order. Next, HfO_2-*x*_ thin films were deposited by rf sputtering at 80 ℃. The chamber was evacuated to a pressure of 1 × 10^−5^ Torr. Since Hf is easily oxidized to HfO_2_, [62] to obtain oxygen-deficient thin films containing Hf suboxide, argon with a partial pressure of 10 mTorr was used as the working gas without any reactive gas, such as oxygen. A nominally stoichiometric ceramic HfO_2_ target of 99.99% purity was used. Finally, a square Au top electrode with dimensions of 70 µm × 70 µm was deposited through thermal evaporation through a metal shadow mask. The thickness of all the samples was measured using a surface profiler (Bruker Dektak XTL).

### Characterization

The current–voltage (*I-V*) characteristics of HfO_2-*x*_ thin films were measured in a probe station using a Keithley 2400 digital source meter. In addition, XPS spectra were obtained with a Sigma Probe (Thermo Fisher Scientific, UK) and were calibrated using the binding energy of the C 1 s peak at 285.0 eV.

Local electrical properties were characterized by KPFM and cAFM using a commercial AFM (n-Tracer, NanoFocus Inc.) with a Pt/Ir-coated tip, PPP-EFM-50 (Nanosensor), for both contact and noncontact modes. Topography and surface potential images were simultaneously obtained. The scan speed was set to 0.5 Hz to minimize any topography-induced artifacts. KPFM signals were obtained by applying an AC voltage of 1.0 V at 61 kHz with an SR830 Lock-in Amplifier. The work function of the Pt/Ir tip was calibrated as 4.6 eV by using a cleaved highly ordered pyrolytic graphite reference sample. Local current maps were obtained by cAFM by applying sample biases up to ± 10 V between the tip and silver bottom electrode.

## Supplementary Information

**Additional file 1: Fig. S1.** Simulation of mean free path of a sputtered atom as a function of the sputtered ion’s kinetic energy. **Fig. S2.** XPS spectra in relatively stoichiometric HfO_2_ thin film. The fitting results for (a) the Hf 4f spectra and (b) O 1s spectra. **Fig. S3.** Initial electrical conductance of the conducting, intermediate, and insulating HfO_2-__*x*_ thin films as a function of the area of Hf suboxide obtained from XPS spectra. The yellow line is a guide for the eye. **Fig. S4.** Topographies (left) and corresponding CPD mapping images (left) of (a) metallic, (b) intermediate, and (c) insulating phases. The scale bar measures 200 nm. **Fig. S5.** Topographies of (a) the conducting, (b) intermediate, and (c) insulating phases and the corresponding current mapping images of the (d) metallic, (e) intermediate, and (f) insulating phases. (g) Root-mean-square (RMS) of roughness of (a) the conducting, (b) intermediate, and (c) insulating phases. The scale bar measures 200 nm. **Fig. S6.** Flexible HfO_2-__*x*_ films on (a) high and (b) low convex angular molds and (c) a concave angular mold. (d) Calculated curvature radius (r) and corresponding strain as a function of central angle (θ). **Fig. S7.** Estimation of the (a) grain boundary depth and (b) contact potential depth of a planar HfO_2-x_ thin film. The green and red crosses indicate crest and trough, respectively. **Fig. S8.** (a) Variation of work function in oxygen deficient HfO_2-x_ thin film as a function of bending cycles. The CPD depth (black) and defect density (blue) at the grain boundary of (b) a planar and (c) a bent structure as a function of the bending cycles. **Fig. S9.** (a) Resistance of the conducting and insulating phases as a function of applied strain. Resistance of (b) the conducting and insulating phases and (c) the HRS and LRS of the intermediate phase as a function of the bending cycle. (d) Unipolar resistive switching behavior as a function of the bending cycle. **Fig. S10.** Band diagram of (a) the planar insulating, (b) conducting, and (c) bent conducting phases in the in-plane direction across a grain boundary. Each phase’s space charge distributions are shown at the top, and the orange and the green squares represent trapped hole density and acceptor density due to oxygen vacancies, respectively. At the bottom, IG and GB denote intergrain and grain boundary, respectively. The thickness of the red lines at the grain boundary indicates the trapped hole density

## Data Availability

Not applicable.

## Abbreviations

PET: Polyethylene terephthalate
PEN: Polyethylene naphthalate
HRS: High resistance state
LRS: Low resistance state
XPS: X-ray photoelectron spectroscopy
KPFM: Kelvin probe force microscopy
CPD: Contact potential difference
AFM: Atomic force microscope
cAFM: Conductive atomic force microscopy
