# Supplementary material for: Grain boundary passivation via balancing feedback of hole barrier modulation in HfO2-x for nanoscale flexible electronics
Source: Nano Converg. 2022 Sep 30;9:43. doi: 10.1186/s40580-022-00336-4 (PMC9525481; doi:10.1186/s40580-022-00336-4)
Supplement: Supplementary file 1 — Additional file 1: Fig. S1. Simulation of mean free path of a sputtered atom as a function of the sputtered ion’s kinetic energy. Fig. S2. XPS spectra in relatively stoichiometric HfO2 thin film. The fitting results for (a) the Hf 4f spectra and (b) O 1s spectra. Fig. S3. Initial electrical conductance of the conducting, intermediate, and insulating HfO2-x thin films as a function of the area of Hf suboxide obtained from XPS spectra. The yellow line is a guide for the eye. Fig. S4. Topographies (left) and corresponding CPD mapping images (left) of (a) metallic, (b) intermediate, and (c) insulating phases. The scale bar measures 200 nm. Fig. S5. Topographies of (a) the conducting, (b) intermediate, and (c) insulating phases and the corresponding current mapping images of the (d) metallic, (e) intermediate, and (f) insulating phases. (g) Root-mean-square (RMS) of roughness of (a) the conducting, (b) intermediate, and (c) insulating phases. The scale bar measures 200 nm. Fig. S6. Flexible HfO2-x films on (a) high and (b) low convex angular molds and (c) a concave angular mold. (d) Calculated curvature radius (r) and corresponding strain as a function of central angle (θ). Fig. S7. Estimation of the (a) grain boundary depth and (b) contact potential depth of a planar HfO2-x thin film. The green and red crosses indicate crest and trough, respectively. Fig. S8. (a) Variation of work function in oxygen deficient HfO2-x thin film as a function of bending cycles. The CPD depth (black) and defect density (blue) at the grain boundary of (b) a planar and (c) a bent structure as a function of the bending cycles. Fig. S9. (a) Resistance of the conducting and insulating phases as a function of applied strain. Resistance of (b) the conducting and insulating phases and (c) the HRS and LRS of the intermediate phase as a function of the bending cycle. (d) Unipolar resistive switching behavior as a function of the bending cycle. Fig. S10. Band diagram of (a) the planar insulating, (b) [file 40580_2022_336_MOESM1_ESM.docx]

**Supplementary Information**

**Grain boundary passivation *via* balancing feedback of hole barrier modulation in HfO_2-_*_x_* for nanoscale flexible electronics**

**Yeon Soo Kim^1^, Harry Chung^1^, Suhyoun Kwon^2^, Jihyun Kim^2^, and William Jo^1,2*^**

^1^New and Renewable Energy Research Center (NREC), Ewha Womans University, Seoul 03760, Korea

^2^Department of Physics, Ewha Womans University, Seoul 03760, Korea

*Correspondence and requests for materials should be addressed to W.J. (email: [wmjo@ewha.ac.kr](mailto:wmjo@ewha.ac.kr)).

**
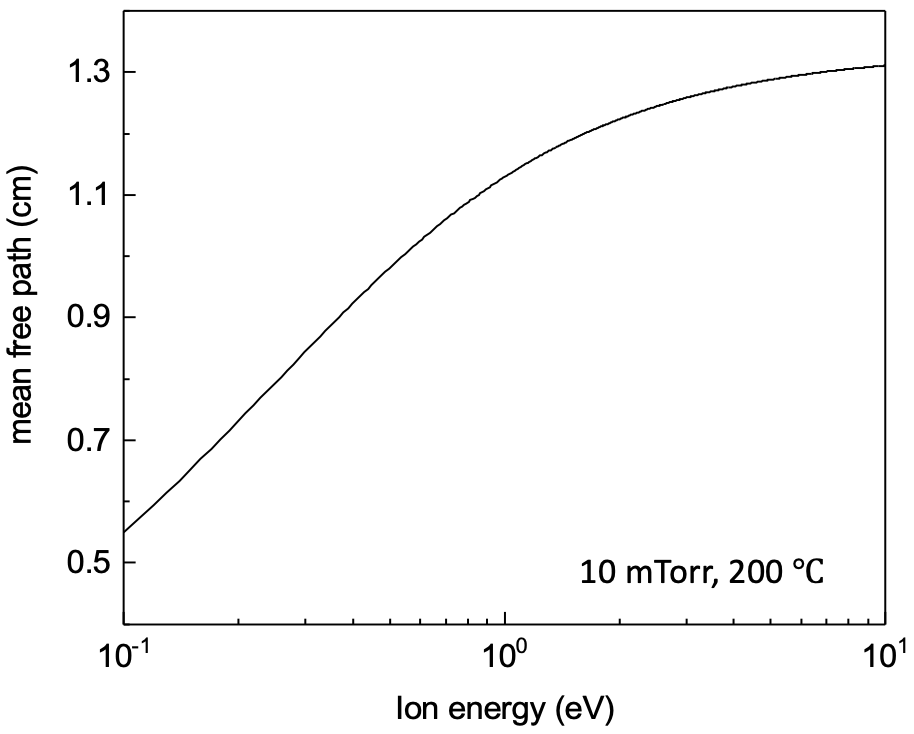
**

**Fig. S1.** Simulation of mean free path of a sputtered atom as a function of the sputtered ion’s kinetic energy.


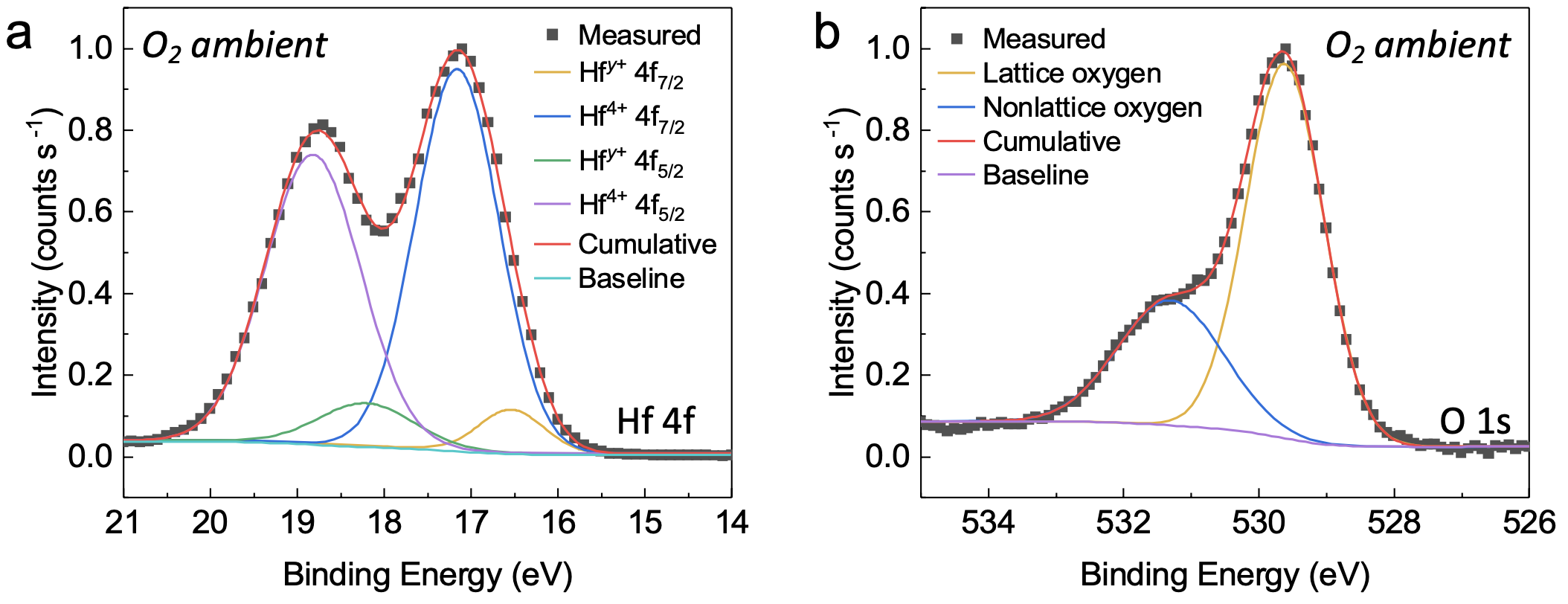


**Fig. S2.** XPS spectra in relatively stoichiometric HfO_2_ thin film. The fitting results for (a) the Hf 4f spectra and (b) O 1s spectra.

**
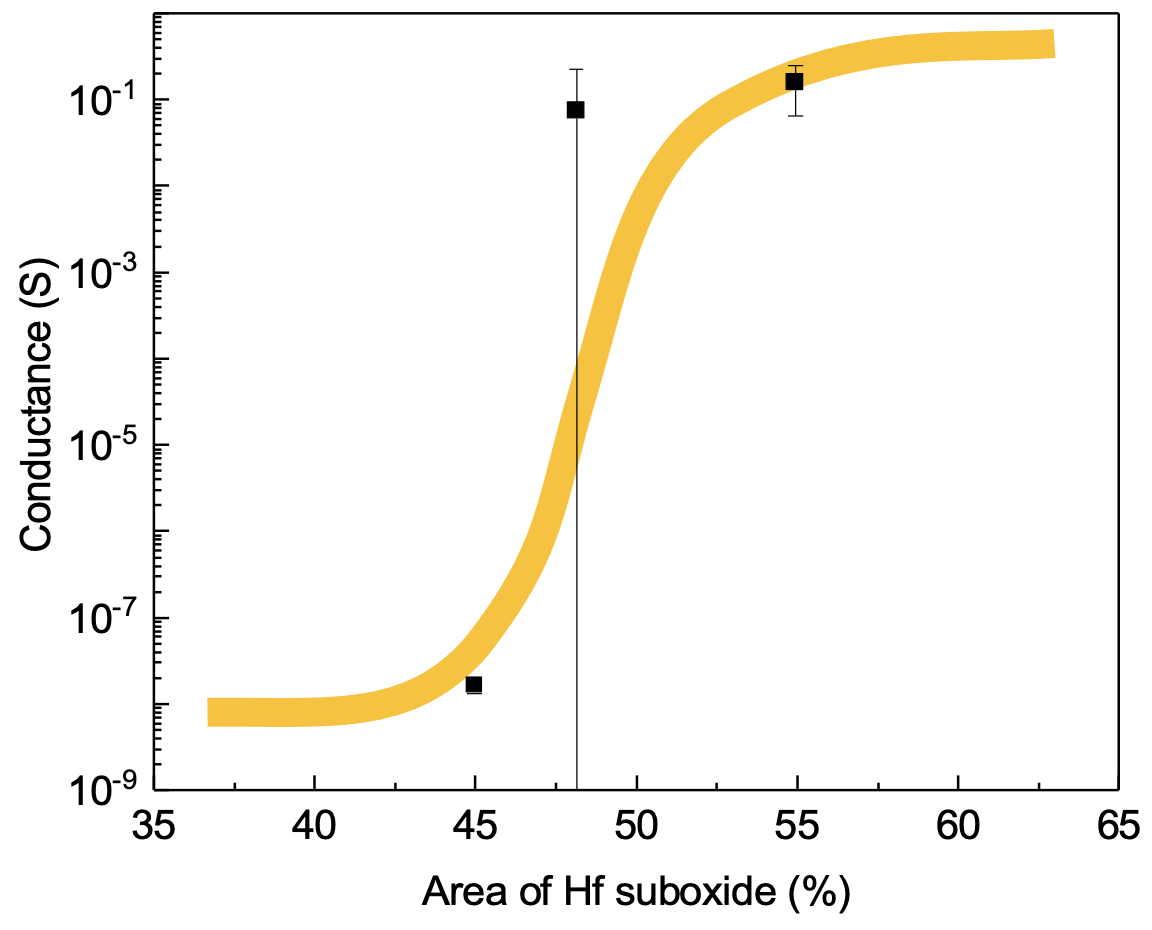
**

**Fig. S3.** Initial electrical conductance of the conducting, intermediate, and insulating HfO_2-_*_x_* thin films as a function of the area of Hf suboxide obtained from XPS spectra. The yellow line is a guide for the eye.


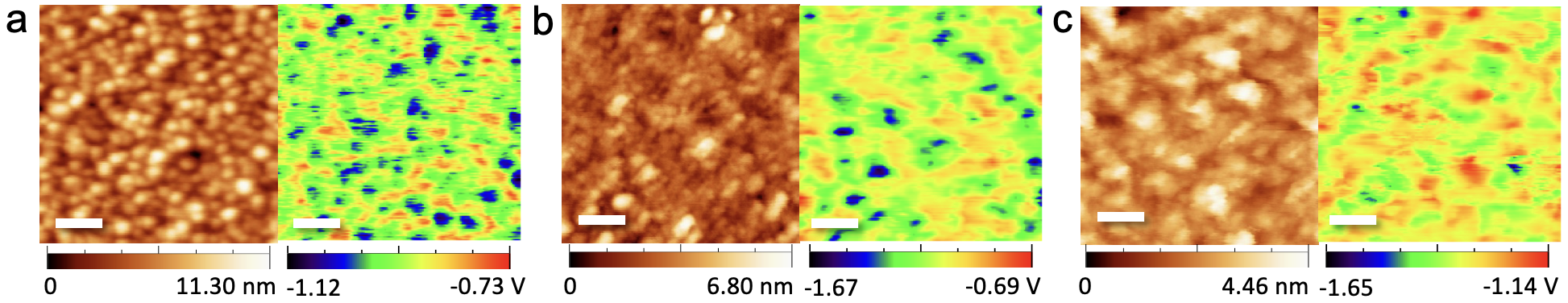


**Fig. S4.** Topographies (left) and corresponding CPD mapping images (left) of (a) metallic, (b) intermediate, and (c) insulating phases. The scale bar measures 200 nm.


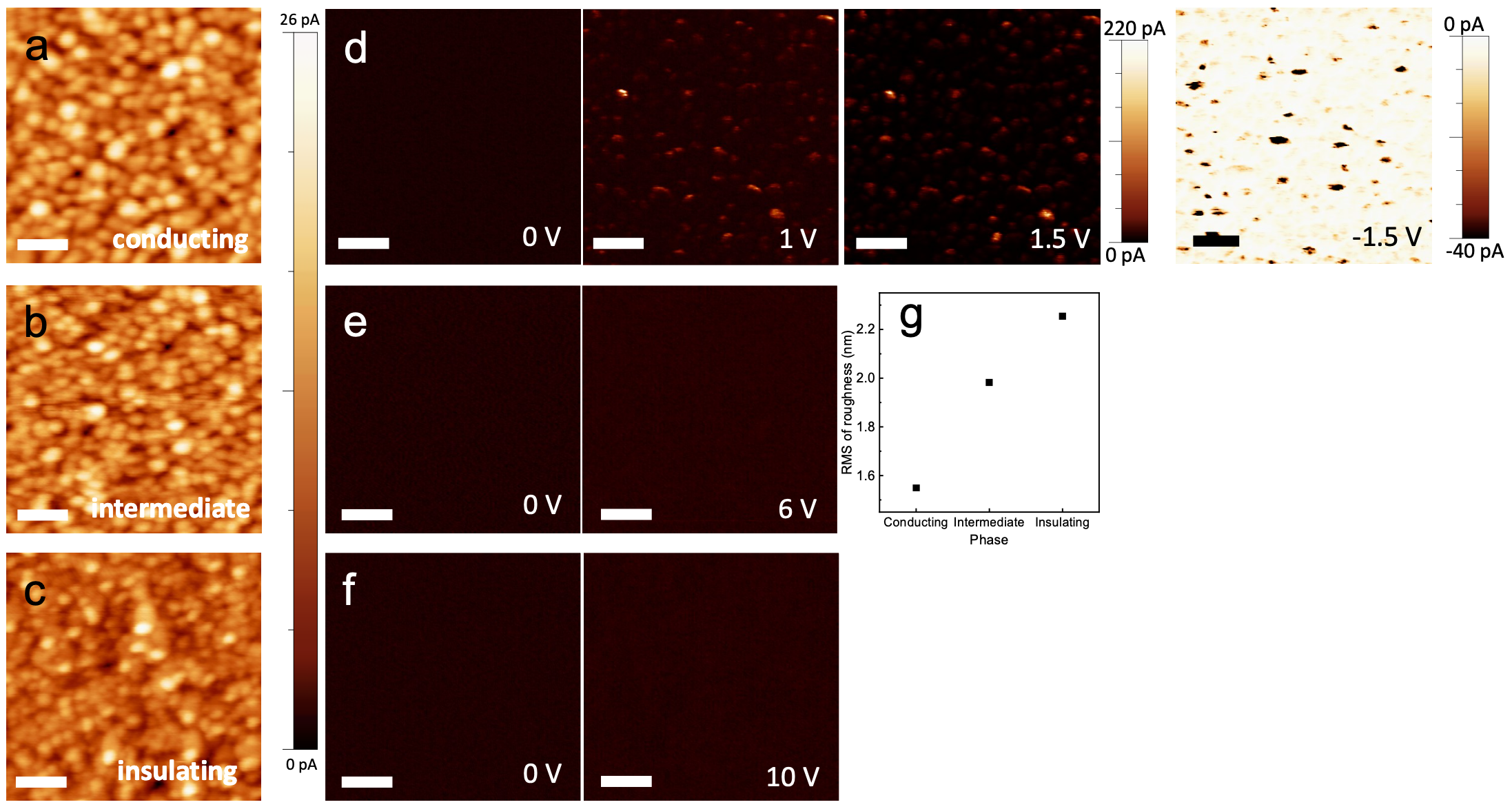


**Fig. S5.** Topographies of (a) the conducting, (b) intermediate, and (c) insulating phases and the corresponding current mapping images of the (d) metallic, (e) intermediate, and (f) insulating phases. (g) Root-mean-square (RMS) of roughness of (a) the conducting, (b) intermediate, and (c) insulating phases. The scale bar measures 200 nm.


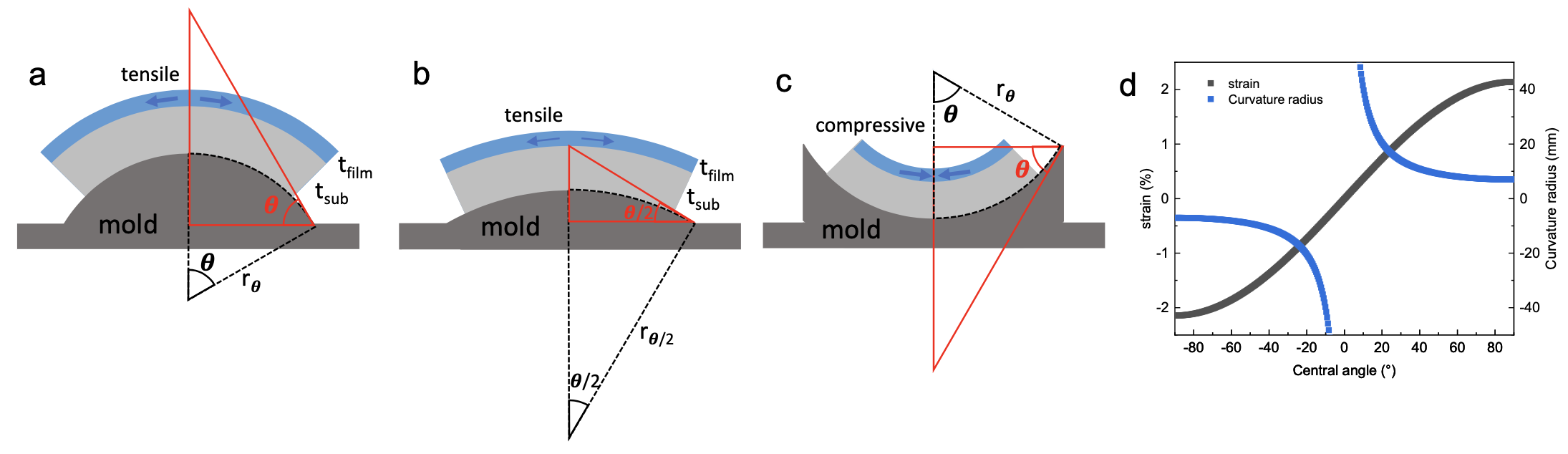


**Fig. S6.** Flexible HfO_2-_*_x_* films on (a) high and (b) low convex angular molds and (c) a concave angular mold. (d) Calculated curvature radius (r) and corresponding strain as a function of central angle (𝜃).


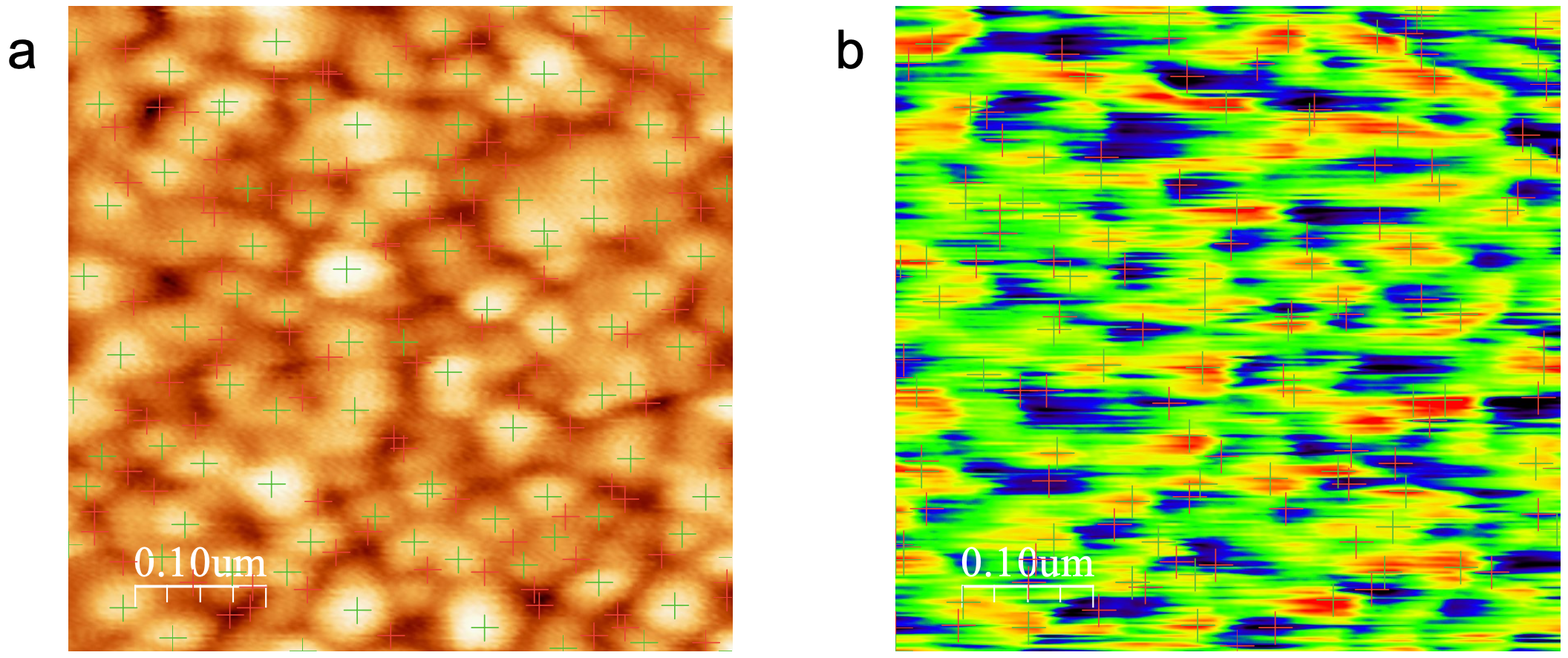


**Fig. S7.** Estimation of the (a) grain boundary depth and (b) contact potential depth of a planar HfO_2-x_ thin film. The green and red crosses indicate crest and trough, respectively.


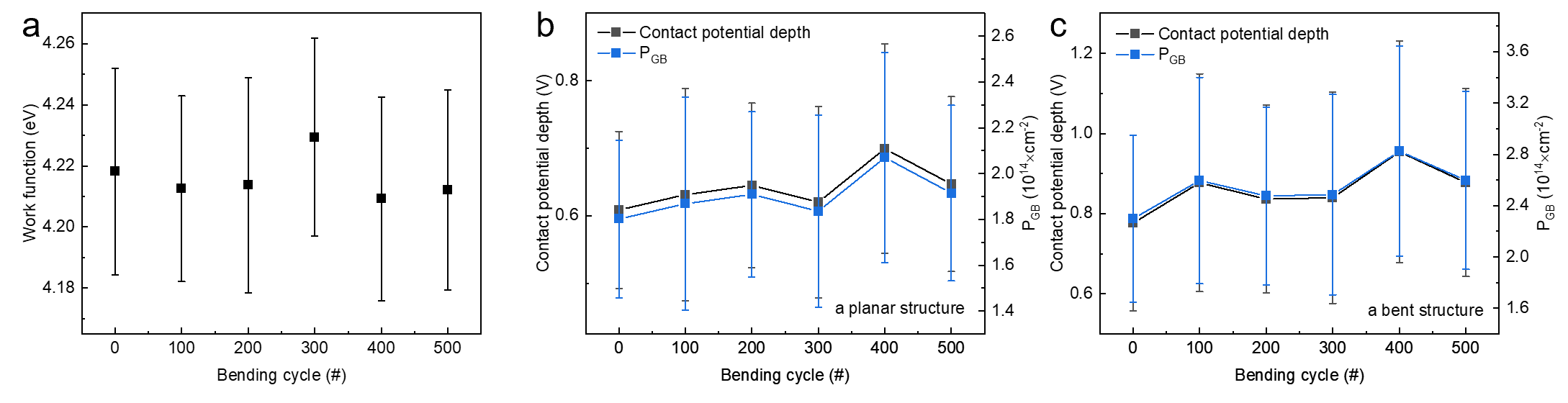
 **Fig. S8.** (a) Variation of work function in oxygen deficient HfO_2-x_ thin film as a function of bending cycles. The CPD depth (black) and defect density (blue) at the grain boundary of (b) a planar and (c) a bent structure as a function of the bending cycles.


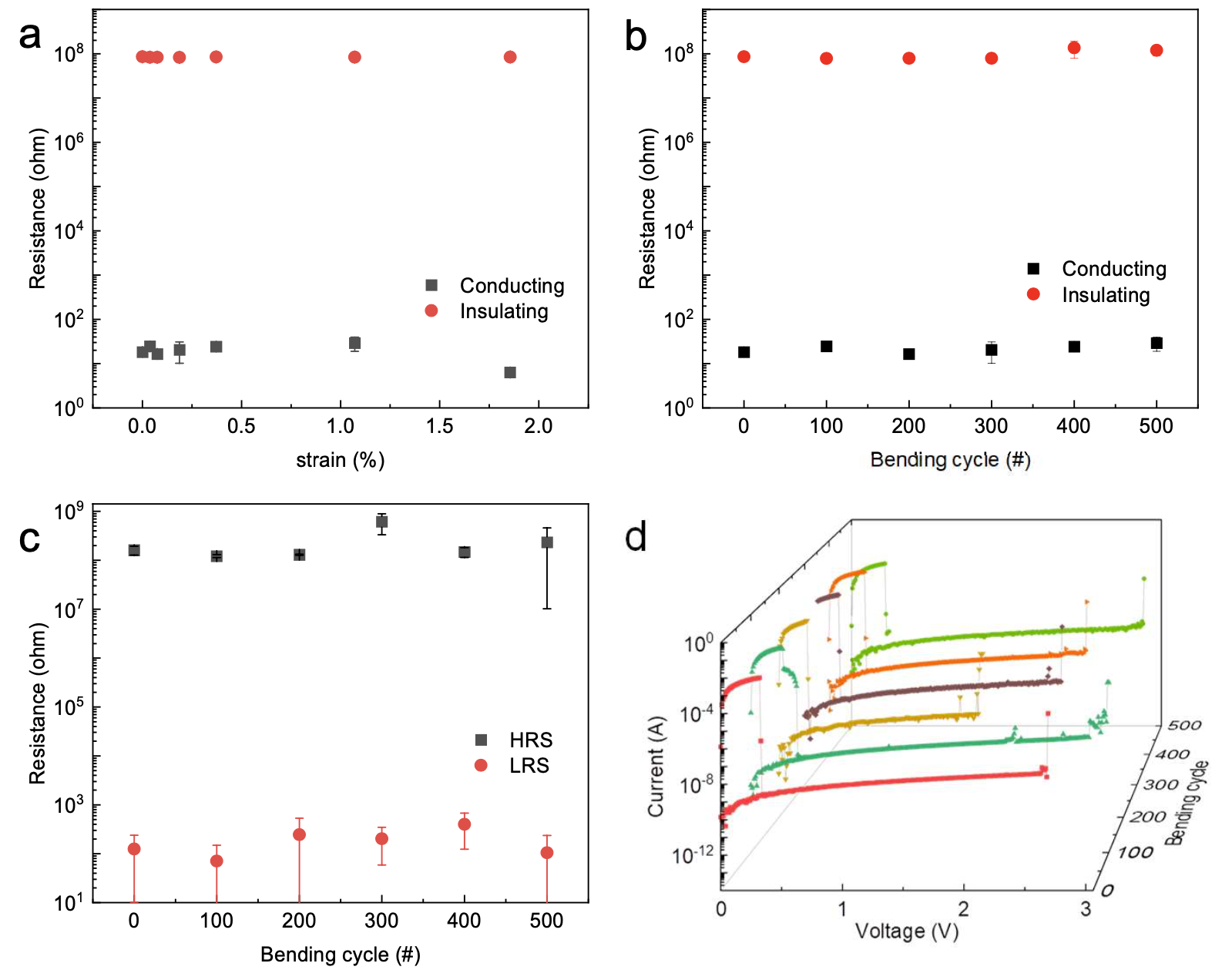


**Fig. S9.** (a) Resistance of the conducting and insulating phases as a function of applied strain. Resistance of (b) the conducting and insulating phases and (c) the HRS and LRS of the intermediate phase as a function of the bending cycle. (d) Unipolar resistive switching behavior as a function of the bending cycle.


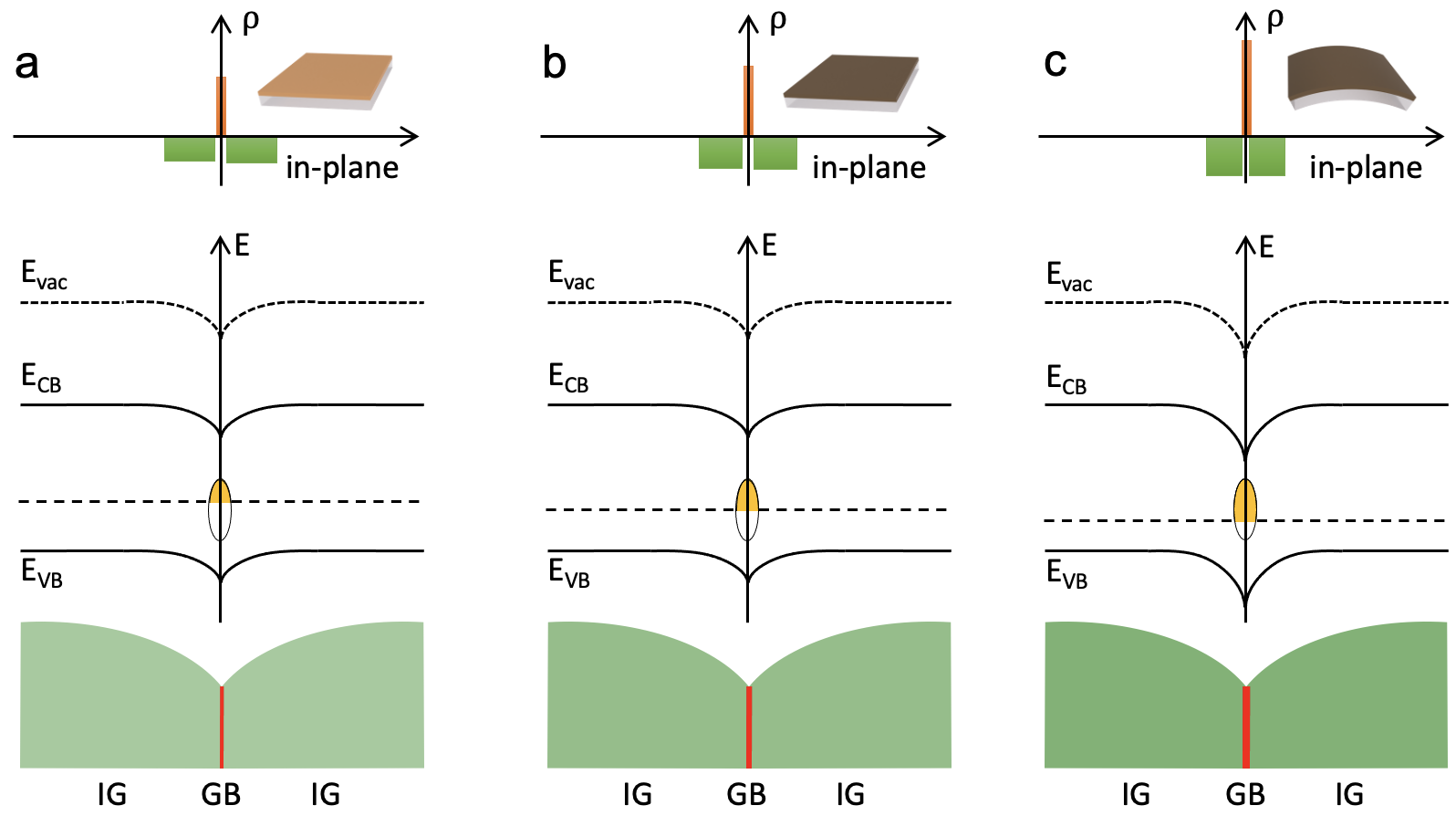


**Fig. S10.** Band diagram of (a) the planar insulating, (b) conducting, and (c) bent conducting phases in the in-plane direction across a grain boundary. Each phase’s space charge distributions are shown at the top, and the orange and the green squares represent trapped hole density and acceptor density due to oxygen vacancies, respectively. At the bottom, IG and GB denote intergrain and grain boundary, respectively. The thickness of the red lines at the grain boundary indicates the trapped hole density.
